# Prevalence of the protective *OAS1* rs10774671-G allele against severe COVID-19 in Moroccans: implications for a North African Neanderthal connection

**DOI:** 10.1007/s00705-024-06038-y

**Published:** 2024-04-25

**Authors:** Fatima Zahra El Yousfi, Abbas Ermilo Haroun, Chaimae Nebhani, Jihane Belayachi, Omar Askander, Elmostafa El Fahime, Hakima Fares, Khalid Ennibi, Redouane Abouqal, Rachid Razine, Ahmed Bouhouche

**Affiliations:** 1 Laboratory of Human Genetics, Medical School and Pharmacy, University Mohammed V in Rabat, Rabat, Morocco; 2 Laboratory of Biostatistics, Clinical and Epidemiological Research, Department of Public Health, Medical School and Pharmacy, University Mohammed V in Rabat, Rabat, Morocco; 3 Laboratory of Community Health, Department of Public Health, Medical School and Pharmacy, University Mohammed V in Rabat, Rabat, Morocco; 4 Acute Medical Unit, Ibn Sina University Hospital, Rabat, Morocco; 5 Faculty of Medical Science, Mohammed VI Polytechnic University, Benguerir, Morocco; 6 Molecular Biology and Functional Genomics Platform, National Center for Scientific and Technical Research, Rabat, Morocco; 7 Intensive Care Department, Cheikh Zaid International Universitary Hospital, Rabat, Morocco; 8 Virology, Infectious and Tropical Diseases Center, Hopital Militaire d’Instruction Mohammed V, Rabat, Morocco; 9 Genomic Center of the Cheikh Zaid Foundation, Abulcasis International University of Health Sciences, Rabat, Morocco

## Abstract

The clinical presentation of COVID-19 shows high variability among individuals, which is partly due to genetic factors. The *OAS1/2/3* cluster has been found to be strongly associated with COVID-19 severity. We examined this locus in the Moroccan population for the occurrence of the critical variant rs10774671 and its respective haplotype blocks. The frequency of single-nucleotide polymorphisms (SNPs) in the cluster of *OAS* immunity genes in 157 unrelated individuals of Moroccan origin was determined using an in-house exome database. *OAS1* exon 6 of 71 SARS-CoV-2-positive individuals with asymptomatic/mild disease and 74 with moderate/severe disease was sequenced by the Sanger method. The genotypic, allelic, and haplotype frequencies of three SNPs were compared between these two groups. Finally, males in our COVID-19 series were genotyped for the Berber-specific marker E-M81. The prevalence of the *OAS1* rs10774671-G allele in present-day Moroccans was found to be 40.4%, which is similar to that found in Europeans. However, it was found equally in both the Neanderthal GGG haplotype and the African GAC haplotype, with a frequency of 20% each. These two haplotypes, and hence the rs10774671-G allele, were significantly associated with protection against severe COVID-19 (*p* = 0.034, *p* = 0.041, and *p* = 0.008, respectively). Surprisingly, in men with the Berber-specific uniparental markers, the African haplotype was absent, while the prevalence of the Neanderthal haplotype was similar to that in Europeans. The protective rs10774671-G allele of *OAS1* was found only in the Neanderthal haplotype in Berbers, the indigenous people of North Africa, suggesting that this region may have served as a stepping-stone for the passage of hominids to other continents.

**Supplementary Information:**

The online version contains supplementary material available at 10.1007/s00705-024-06038-y.

## Introduction

COVID-19, caused by the coronavirus SARS-CoV-2, appeared in November 2019 in Wuhan, Hubei province, China, before spreading around the world and causing a pandemic [1, 2]. The consequences of SARS-CoV-2 infection vary greatly from person to person and from population to population. While most infected individuals are asymptomatic or minimally symptomatic, some develop severe or even critical illness. Older people and those with underlying medical condition, such as cardiovascular disease, diabetes, chronic respiratory disease, or cancer, are at greater risk of developing a severe form [3]. However, these risk factors alone do not explain the variability in severity observed. These differences could partly result from individual genetic susceptibility. The scientific community has been mobilized to respond to the health crisis to identify the genetic determinants of COVID-19 that protect people from or predispose them to severe manifestations of the disease. A genetic study conducted on patients of European ancestry identified a risk locus for severe COVID-19 in the 2’-5’-oligoadenylate synthetase 1/2/3 (*OAS1/2/3*) gene cluster [4]. Subsequently, it was reported that elevated levels of OAS1 in the plasma were associated with reduced COVID-19 susceptibility and severity [5].

This genetic region of chromosome 12q24.13 carries a haplotype of approximately 75 kb of Neanderthal origin [6], in which the discovery of the causal variant was possible through studies conducted on a multiancestry cohort including patients from both African and European ancestry. It consists of the splice-acceptor site of exon 6 in *OAS1* (rs10774671 A>G) [7, 8]. In humans, *OAS1* is alternatively spliced to produce five isoforms, of which p42 and p46 are the major forms. The G allele of the rs10774671 SNP specifically produces the p46 isoform, whereas the A allele predominantly encodes the p42 isoform. The p46 has a prenylation signal at its C-terminus that localizes the protein to the double-membrane intracellular vesicles where SARS-CoV-2 replicates, leading to recognition of viral RNA and subsequent activation of RNase L for virus inhibition [9–12]. Moreover, *OAS1* SNPs have been reported to be associated with susceptibility to West Nile virus infections [13] and to be involved in altered cellular function, leading to several autoimmune and infectious diseases, suggesting that the OAS genes play a critical role in the innate immune response to viruses [14–18].

In this study, we assessed the prevalence of several variants at the *OAS* gene cluster that comprise the Neanderthal haplotype by analyzing exome data of 157 unrelated Moroccan individuals and observed that the *OAS1* rs10774671-G allele was found in both Neanderthal and African haplotypes. We then investigated the rs10774671 variant in 146 Moroccan volunteers who were positive for SARS-CoV-2 infection and found that it was significantly associated with COVID-19 severity. Interestingly, in Berbers of both paternal and maternal lineages, the African haplotype containing the G allele was absent, while the frequency of Neanderthal-derived haplotypes was similar to that in Europeans.

## Materials and methods

### Frequency of OAS gene variants in the Moroccan population

To assess the frequency in the Moroccan population of the Neanderthal haplotype in the cluster of *OAS* immunity genes in the chromosomal region 12q24.13, we analyzed exome data from 157 unrelated patients suspected of having a rare hereditary disease. These patients were recruited from the Department of Neurology of Specialties Hospital and Cheikh Zaid Hospital of Rabat. Part of the data were obtained from an in-house exome database, and the rest were obtained by whole-exome sequencing (WES), which was carried out as part of a collaborative project with 3Billion (Seoul, Korea). Data were collected for variants rs1131454, rs10774671, rs1131476, rs1051042, and rs2660 in *OAS1*, rs1859330, rs1859329, and rs2285932 in *OAS3*, and the variant rs1293767 in *OAS2*. These variants comprise the Neanderthal haplotype at the *OAS* locus, as described elsewhere [19].

### Recruitment of COVID-19 patients

To determine whether *OAS* gene variants were associated with the severity of COVID-19 in the Moroccan population, it was essential to minimize the effects of underlying medical conditions such as comorbidities by reducing the upper age limit for inclusion of patients in the study. Thus, we limited the study cohort to include males or females of Moroccan origin, aged between 18 and 65 years old, with a positive RT-PCR test for SARS-CoV-2, and who had not been vaccinated against the virus. Pregnant patients were excluded from the study. A total of 146 COVID-19 patients were enrolled during a period of the pandemic that was dominated by the Alpha and Beta variants of SARS-CoV-2. All participants filled out a form to provide socio-demographic data for the establishment of their ethnicity and medical history, including comorbidities, if applicable. Patients were recruited by three different hospital centers involved in a national COVID-19 research consortium, including a medical emergency department (CHU Ibn Sina, Rabat), a virology department (Military Hospital, Rabat), and an intensive care unit (Cheikh Zaid International University Hospital, Rabat). Outcomes were defined as asymptomatic (without symptoms), mild (non-hospitalized), moderate (hospitalized but not requiring invasive ventilation), and severe (hospitalized requiring invasive ventilation or death). The 146 patients were grouped into the following severity categories: asymptomatic (*N* = 21), mild COVID-19 (*N* = 50), moderate COVID-19 (*N* = 48), and severe COVID-19 (*N* = 27). Asymptomatic and mild patients were grouped together (*N* = 71) and compared to the combined group of moderate/severe patients (*N* = 75). Five ml of venous blood was collected in an EDTA tube from each participant and stored at -20°C until DNA extraction. Written informed consent was signed by all patients before their enrollment. This study was performed in accordance with the principles of the Declaration of Helsinki. Approval was granted by the Ethics Committee of Cheikh Zaid Hospital of Rabat (ID: CEFCZ/AB/PR_RFG).

### Genetic analysis

Genomic DNA (gDNA) of 146 SARS-CoV-2-positive volunteers was extracted using a MagPurix Blood DNA Extraction Kit with a Zinexts MagPurix EVO 24 CE IVD system (Zinexts Life Science Corp., New Taipei City, Taiwan). The quality and concentration of DNA samples was determined using a NanoDrop One Spectrophotometer (Thermo Fisher Scientific). PCR amplification, covering *OAS1* exon 6 and its intron boundaries, was performed using two pairs of specific primers (forward, 5’-GTGGCCAGGCTTCTATACCC-3’; reverse, 5’-TGGAGTGTGCTGGGTCTATG-3’), which were designed using Primer3 Input (V 0.4.0). PCR amplification was performed using 100 ng of template gDNA, 1 µL of each primer (10 µM), 5 µL of 5X MyTaq reaction buffer, and 0.2 µl of MyTaq DNA polymerase (Bioline) in a total reaction volume of 25 µL. Thermal conditions were as follows: 95°C for 3 min, 35 cycles of 95°C for 5 s, 60°C for 15 s, and 72°C for 10 s, with a final extension at 72°C for 10 min. The PCR products were visualized by 1.5% agarose gel electrophoresis. Five microliters of PCR amplicons were purified using ExoSap-IT Express Reagent, and 2 microliters of the product was used for cycle sequencing, using a BigDye Terminator v3.1 Ready Reaction Cycle Sequencing Kit. Electrophoresis was performed on a SeqStudio Genetic Analyzer, and sequence data were analyzed using SeqScape 4 software (Thermo Fisher Scientific).

The genetic marker E-M81, which has been reported to be specific to the male lineage of autochthonous Berbers of North Africa, was analyzed in all 80 males in the COVID-19-positive cohort of patients. The marker was amplified by PCR and sequenced by the Sanger method as described previously [20].

### Statistical analysis

Statistical analysis was performed using jamovi software (version 1.6). The evaluation of demographic variables and disease characteristics was done using descriptive statistics. A chi-square (χ^2^) test was used before the association tests to assess deviation from Hardy-Weinberg equilibrium (HWE) in the control group. The genotypic and allelic frequencies were compared between patients and controls to assess the associations of the studied SNPs with COVID-19 severity. The χ^2^ test was used to identify differences in the genotypic distribution between study groups under an additive genetic model. For allelic and haplotype models, odds ratios (ORs) at 95% confidence intervals (CI) were estimated using logistic regression analysis, and a *p*-value less than 0.05 was considered statistically significant.

## Results

### Frequency of Neanderthal alleles in the OAS gene cluster in Moroccans

**Fig. 1:**
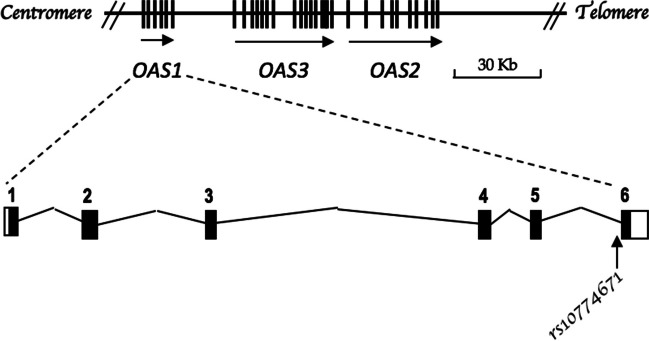
OAS gene cluster at chromosome 12q24.13 and structural organization of *OAS1*, showing the location of the splice variant rs10774671

The genotypes of nine SNPs in the cluster of *OAS* immunity genes in the chromosomal region 12q24.13 (Fig. 1) were extracted from an exome sequencing database of 157 unrelated individuals, all of Moroccan origin. As shown in Table 1, the overall frequencies of these SNPs in Moroccans were found to be close to the minor allele frequency (MAF) of the 1000 genome project. Focusing on *OAS1* variants, the reference G allele of the *OAS1* splice variant rs10774671 was found in 40.4% of the Moroccan individuals tested, which is similar to that in Europeans (35.2%), but much lower than that in Africans (64.0%) and much higher than that in Asians (27.2%). The frequencies of the missense variants rs1131476 and rs1051042 and the 3’UTR rs2660 variant were all identical (21.1%), indicating that they are in complete linkage disequilibrium, which was also the case in the other populations of European, Asian, and African ancestry (Table 1).

**Table 1:** SNP frequencies of the *OAS* locus in 157 individuals of Moroccan origin in comparison with those of the 1000 genomes project

	OAS1					OAS3			OAS2
	rs1131454	rs10774671	rs1131476	rs1051042	rs2660	rs1859330	rs1859329	rs2285932	rs1293767
	c.484G>A	c.1039-1G>A	c.1054G>A	c.1082G>C	c.*84G>A	c.53G>A	c.117C>T	c.1314T>C	c.448+41C>G
	p.Gly162Ser	Splice site	p.Ala352Pro	p.Arg361Met	3'UTR	p.Arg18Lys	p.Ala39=	p.Ile438=	Intronic
Minor allele frequency	0. 470	0.390	0.210	0.210	0.210	0.340	0.210	0.160	0.150
African ancestry	0. 851	0.640	0.027	0.027	0.027	0.446	0.011	0.010	0.011
Asian ancestry	0.414	0.272	0.271	0.271	0.271	0.268	0.268	0.156	0.130
European ancestry	0.425	0.352	0.345	0.345	0.345	0.370	0.365	0.328	0.325
Moroccan population	0.554	0.404	0.210	0.210	0.210	0.283	0.213	0.197	0.194

**Fig. 2:**
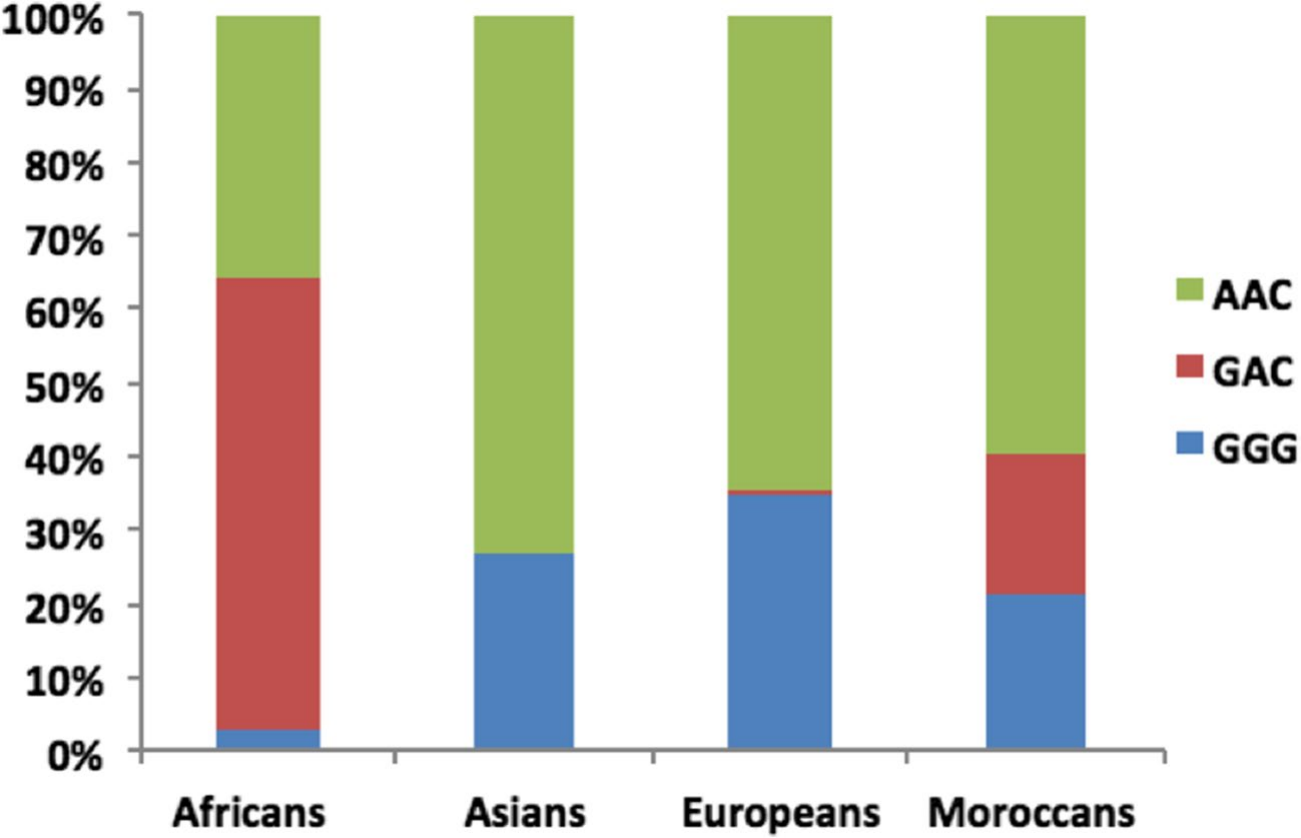
Prevalence of the three possible haplotypes with the three *OAS1* SNPs studied in 157 individuals of Moroccan origin in comparison with the data of the 1000 Genome Project of Africans, Asians, and Europeans

We then investigated the haplotypes formed by the SNPs rs10774671, rs1131476, and rs1051042, which allowed the predominantly protective Neanderthal-derived haplotype to be identified in this genetic region. The haplotypes consisting of these three SNPs were GGG, GAC, and AAC, and a graphical representation of their distribution in present-day Moroccans in comparison with people of different ancestry is shown in Fig. 2. The *OAS1* rs10774671-G allele in Moroccans was included in both Neanderthal GGG and African GAC haplotypes, with frequencies of 21% and 19.4%, respectively. In comparison to other ancestries, the GGG haplotype accounts for 34.5% of Europeans, 27.1% of Asians, and only 2.7% of Africans. The GAC haplotype is predominant in Africans, however, with a frequency of 61.3%, and is almost absent in Europeans and Asians (0.7% and 0.001%, respectively). Finally, the risk haplotype AAC was the prevalent haplotype in the Asian population, with 72.4% (Table 1).

### Association study in Moroccan COVID-19 patients

The demographic and clinical data of the two studied groups are shown in Table 2. The mean age and standard deviation of patients with asymptomatic/mild COVID-19 was 44.1 ± 13.5 years, which was significantly lower than that of patients with moderate/severe COVID-19, which was 51.4 ± 10.5 years (*p* < 0.001). There was no significant difference in the sex ratio between the two groups (OR = 1.04,* p* < 0.897), with a female-to-male ratio of approximately 1:1. As expected, the asymptomatic/mild group displayed a significantly lower frequency of comorbidity than in the moderate/severe group (OR = 2.85, *p* < 0.004), with 22.5% and 56%, respectively. Likewise, the mortality rate was 0% in the asymptomatic/mild group and 8% in the moderate/severe group, and this difference was statistically significant (OR = 0.07, *p* < 0.015).

**Table 2:** Demographic information about the two groups of COVID-19 patients

	Disease severity				OR (CI 95%)	*p*-value
	Asymptomatic/mild (*n* = 71)		Moderate/severe (*n* = 75)			
	No.	%	No.	%		
Age (y) mean ± SD	44.1 + 13.5		51.4 + 10.5			< .001
Sex						
Male	39	54.9	41	54.7	1.04 (0.544-2.01)	0.897
Female	32	45.1	34	45.3		
Comorbidity	16	22.5	42	56.0	2.85 (1.39-5.85)	0.004
Mortality	00	0.0	06	8.0	0.07 (0.004-1.35)	0.015

The distribution of genotypes, alleles, and haplotypes of the three *OAS1* SNPs according to severity are presented in Table 3. Among the 292 alleles analyzed, the ancestral G allele represented 40% for the rs10774671 variant and 21.23% for both the rs1131476 and rs1051042 variants. The comparison between the asymptomatic/mild and moderate/severe groups showed a significant difference under both the additive (*p* = 0.014) and allelic (OR 0.528 [95% CI 0.329-0.848], *p* = 0.008 models). However, no statistically significant differences in the distribution of the rs1131476 polymorphism were observed between the two groups under either the additive (*p* = 0.248) or allelic (OR 0.617 [95% CI 0.350-1.090], *p* = 0.096) model, suggesting that this missense mutation was not associated with COVID-19 severity in Moroccans.

**Table 3:** Comparison of the genotypic and allelic distribution of the *OAS1* rs10774671 and rs11314476 SNPs between the asymptomatic/mild and moderate/severe groups of COVID-19 patients

SNP	Genetic model		Asymptomatic/mild (*N* = 71)		Moderate/severe (*N* = 75)		*p*	OR (95% CI)
			*N*	*%*	*N*	*%*		
rs10774671		GG	18	25.3	06	08.0		
	Additive	GA	32	45.1	37	49.3	**0.014**	
		AA	21	29.6	32	42.7		
	Allelic	G	68	47.9	49	32.7	**0.008**	**0.528 (0.329 – 0.848)**
		A	74	52.1	101	67.3	Ref	
rs11314476		GG	05	07.0	02	02.7		
	Additive	GA	26	36.6	22	29.3	0.248	
		AA	40	56.4	51	68.0		
	Allelic	G	36	25.4	26	17.3	0.096	0.617 (0.350 – 1.090)
		A	106	74.6	124	82.7	Ref	

Values in bold indicate a significant difference

A comparison of the occurrence of these polymorphisms in their respective haplotype block between the two groups is shown in Table 4. The three selected SNPs formed only three haplotypes GGG, GAC, and AAC. The haplotypes GGG and GAC were common in both asymptomatic/mild and moderate/severe patients and were associated when compared to the risk AAC haplotype, with OR 0.529 [95% CI 0.285 – 0.973], *p* = 0.034 and OR 0.527 [95% CI 0.028 – 0.509], *p* = 0.041, respectively. These two haplotypes thus confer protection against severe COVID-19 when compared to the risk haplotype AAC in patients of Moroccan origin.

**Table 4:** Comparison of the haplotype distribution consisting of rs10774671, rs1131462, and rs1051042 between the asymptomatic/mild group and the moderate/severe group of COVID-19 patients

	Asymptomatic/mild (*N* = 71)		Moderate/severe (*N* = 75)		*p*	OR (95% CI)
	*N*	*%*	*N*	*%*		
Haplotype						
GGG	36	25.4	26	17.3	**0.034**	**0.529 **(0.285 – 0.973)
GAC	32	22.5	23	15.3	**0.041**	**0.527** (0.028 – 0.509)
AAC	74	52.1	101	67.4	Ref	

Values in bold indicate a significant difference

### Distribution of OAS1 haplotypes in Berbers

**Fig. 3:**
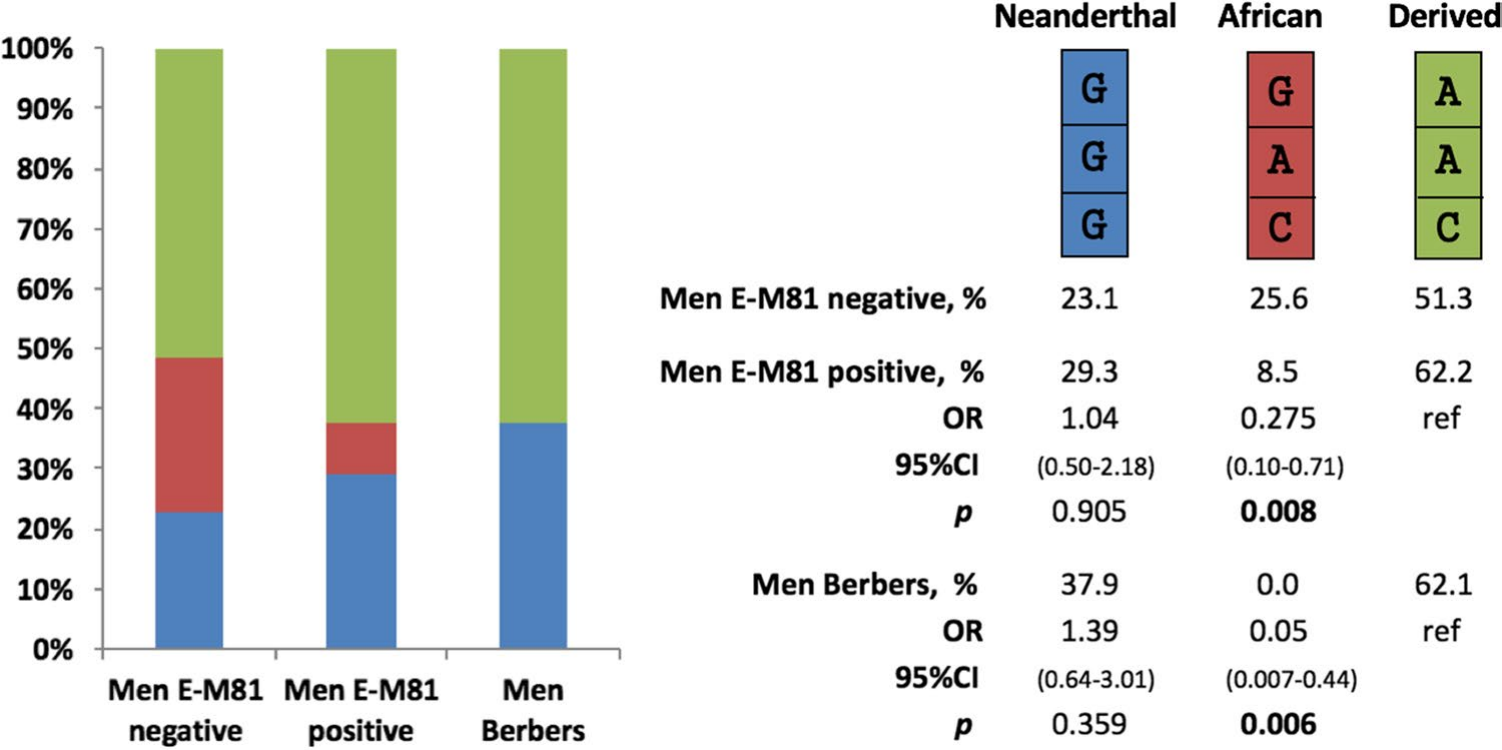
Frequencies of the three haplotypes consisting of the three *OAS1* SNPs studied in 39 males who were negative and 41 males who were positive for the E-M81 marker, all of whom were positive for SARS-CoV-2, as well as 29 male *LRRK2* G2019S carriers whose Berber origin had been established for both the paternal and maternal lineages. The groups were compared using logistic regression.

Among the 80 males in our cohort of SARS-CoV-2-positive individuals, 41 were positive and 39 were negative for the E-M81 marker. The distribution of the haplotypes GGG, GAC, and AAC in these two groups, as well as in 29 males with Parkinson's disease carrying the *LRRK2* G2019S mutation whose Berber origin had been established for both paternal and maternal lineages [20] are presented in Fig. 3. The results showed that the prevalence of the African GAC haplotype in males with the E-M81 marker was only 8.5%, which was significantly lower than that of males who were negative for this marker, who had a prevalence of 25.7% (OR = 0.275 (0.10-0.71) and *p* = 0.008). Furthermore, the GAC haplotype was completely absent in males who were of Berber origin in both their maternal and paternal lineages (OR = 0.05 (0.007-0.44) and *p* = 0.006). The prevalence of both the GGG and AAC haplotypes in these purely Berber males was similar to that found in individuals of European ancestry.

## Discussion

The clinical presentation of COVID-19 following SARS-CoV-2 infection has shown high interindividual variability, ranging from asymptomatic to death, and this is partly due to genetic factors. The chr12q24.13 region containing the *OAS* gene cluster was recently reported to be strongly associated with COVID-19 severity and mortality [4], and the rs10774671 splice variant was confirmed to be the functionally critical variant of this chromosomal region that influences the outcomes of SARS-CoV-2 infection [7, 8]. The *OAS1* rs10774671-G allele has been shown to increase levels of the p46 isoform, and thus, the circulating levels of the OAS1 protein are strongly associated with a reduced risk of severe COVID-19 [5]. This protective G allele is present in about 35% of individuals of European ancestry and was found in a haplotype inherited from Neanderthals, and it is present in 64% of the African population independently of this Neanderthal-derived haplotype [6].

To our knowledge, there are no data on the prevalence of *OAS* gene cluster variants in the North African population, which, due to the geographical position on the African continent, could provide relevant information on how this G allele at rs10774671 was inherited from the ancestral population common to both modern humans and Neanderthals. Our study thus presents a first insight into this chromosomal region in the Moroccan population, which is culturally, historically, and anthropologically similar to the other neighboring countries of the North African subcontinent. We found that the frequencies of *OAS* cluster SNPs in Moroccans were close to the MAF of the 1000 genome project, in which the *OAS1* rs10774671-G allele represented 40.4%. However, analyzing the haplotypes consisting of three *OAS1* variants that captured the Neanderthal haplotype showed that the rs10774671-G allele was found with equal frequency in both the Neanderthal GGG haplotype and the African GAC haplotype. This distribution appears to be different from that of the European population, in which the G allele was mostly found, at a frequency of 35%, in the Neanderthal haplotype, and from the African population, in which it was mostly found, at a frequency of 64%, in a specific African haplotype, as reported before [6–8].

Our study of three selected SNPs in a cohort of 146 SARS-CoV-2-positive individuals of Moroccan origin who were less than 65 years of age showed that only the splice variant rs10774671 was significantly associated with the severity of COVID-19. Both the AA genotype and the A allele were predictive of severe COVID-19 disease, supporting previous findings that this is the only variant of this locus that influences COVID-19 outcomes [7, 8]. However, although the prevalence of the protective allele *OAS1* rs10774671-G in the Moroccan population was similar to that in Europeans, the severity and mortality caused by SARS-CoV-2 infection are much higher in individuals of European ancestry, even leading to a reduction in life expectancy [21]. This suggests that other genetic factors as well as general health factors and conditions, all interacting in complex ways, are likely to be involved in the observed difference between these two populations.

Both the Neanderthal GGG and African GAC haplotypes containing the rs10774671-G allele were found to be protective against severe COVID-19. Overall, in the 146 individuals who were positive for SARS-CoV-2, the frequencies of the G allele and of the two GGG and GAC haplotypes were similar to those of the control cohort of our WES database, thus confirming an equal prevalence of around 20% each for the Neanderthal and African haplotypes in present-day Moroccans. The presence of the African haplotype could certainly be due to a recent admixture with sub-Saharan African populations, but the chronology of the presence of the Neanderthal haplotype in this population would be difficult to determine precisely.

The Moroccan population is known to be a heterogeneous population having received influences from the south, north, and east, and whose ancient origin was essentially indigenous Berber. To investigate the origin of the Neanderthal haplotype in North Africa, the distribution of the three haplotypes was compared in our cohort of SARS-CoV-2-positive individuals between males with and without the marker E-M81, which is known to be specific to the male lineage of autochthonous Berbers of North Africa. The frequencies of the African GAC haplotype decreased to 8.5% in favor of the Neanderthal GGG haplotype, which increased to 29.3% in males with the E-M81 marker compared to males without this marker, at a frequency of 23% and 25.7%, respectively. GAC haplotypes in males with the E-M81 marker are likely to be derived from the maternal lineage. Surprisingly, the African GAC haplotype was completely absent in males with both paternal and maternal Berber lineages, and the frequencies of the two remaining haplotypes, GGG and AAC, were similar to those in populations of European ancestry.

These results provide evidence for the presence of the Neanderthal protective haplotype among Berbers, who were the ancient indigenous inhabitants of North Africa over the last 10,000 years. However, it is unknown whether this haplotype was imported to North Africa in the “back to Africa” migration or exported to Europe and Asia in the earlier “out of Africa” migration. Several recent pieces of scientific evidence lean towards the second scenario. First, it has been reported that present-day North Africans share the majority of their ancestry with Europeans and West Asians rather than sub-Saharan Africans [22–24]. Along the same lines and in a slightly earlier period, a genetic study of human fossils from the Taforalt region dating back 15,000 years excluded gene flow from southern Europe into northern Africa, as well as a genetic affinity with early Holocene Near Easterners, suggesting a late Pleistocene connection between North Africa and the Near East [25]. However, due to the absence in this region from ancient genomic data at a similar time, the authors could not define the epicenter or the direction of this connection.

In a study of the G2019S mutation of the *LRRK2* gene, a founder-effect mutation found in the haplotype 1 worldwide, but with varying prevalence, was deduced to have originated from a Near-Eastern founder at least 4000 years ago and to have been reintroduced by recurrent gene flow to European and North-African populations [26]. Using uniparental markers, we subsequently showed that the mutation arose in a Berber founder and that its epicenter was indeed in North Africa, where the prevalence was the highest (40%), and that the gene flow by migration took place in the opposite direction [20].

In the same way, the site of Jebel Irhoud located in Morocco has provided valuable information that was missing in the puzzle of the history of hominids and, in particular, their first exit from Africa to other continents. Irhoud hominids were originally considered North African *H. sapiens* interbred with Neanderthals, an African form of Neanderthals, or a North African archaic population [27, 28]. Recently, Hublin et al. [29] thermoluminescently dated the Irhoud fossils to 315,000 years ago, making Jebel Irhoud the oldest site of African hominids with features of anatomically modern humans but more-primitive cranial morphology. Phylogenetic modeling of cranial morphology showed phenotypic similarities of Irhoud fossils to both Neanderthals and early *H. sapiens*, suggesting that they had been introgressed into Neanderthals at the end of the Middle Pleistocene, contributing to the evolution of the Neanderthals [30]. Subsequently, we determined the complete genome sequences of three present-day Moroccans and identified over 200,000 SNPs that were absent from the 1000G and gnomAD databases, suggesting that the Moroccan population has more genetic variability and is therefore older than the European population. Principal component analysis showed that Moroccan genomes form a distinct population that is positioned between the European and African 1000G populations [31].

Sánchez-Quinto et al. [32] analyzed 780,000 SNPs in individuals from different locations in North Africa, including Morocco, and showed that North African populations have a significant excess of alleles shared with Neanderthals, whereas sub-Saharan populations were not affected by this admixture event. Those authors also showed that Neanderthal genetic signals were stronger in populations with a local, pre-Neolithic North African ancestry, confirming that this ancient admixture was not due to recent Near Eastern or European migrations. The first Neanderthal fossil that was discovered was at the Jbel Irhoud site [33], where Jean-Jacques Hublin et al. [29] estimated the fossils to be 300,000 years old, and they were accompanied by a Mousterian-type lithic industry, a lithic culture that has long been associated only with Neanderthals in Eurasia. However, the oldest lithic remains discovered in Morocco belong to the Acheulean and have been dated to around 1.3 million years ago [34, 35]. These data therefore constitute genomic, fossil, and lithic evidence of the presence of Neanderthals in Morocco and their local contributions to anatomically modern humans, reinforcing the idea of a local introgression of the *OAS1* rs10774671-G allele.

Although Africa is acknowledged to be the cradle of humanity, the evolution of the hominid ancestors of *Homo sapiens* did not occur in a linear way within this continent, but rather through a process dictated by severe geographical and climatic conditions. Therefore, the evolution of hominids in North Africa, isolated to the south by a largely arid and desert region, would have occurred separately from that of hominids in sub-Saharan Africa and would probably be at the origin of the initial dispersal out of Africa. Their conquest of the other continents would have been possible via the Strait of Gibraltar during the Ice Age, and later along the Mediterranean via the Fertile Crescent. Answers to questions pertaining to this scenario are most likely to be found by whole-genome sequencing of a large number of present-day individuals and examination of hominid fossils from North Africa.

## Conclusion

The rs10774671-G allele of *OAS1* confers protection against severe COVID-19 in the Moroccan population. Surprisingly, this protective allele was found in the Neanderthal haplotype in Berbers, the indigenous people of North Africa, suggesting that this subcontinent could have been the stepping-stone for the passage of the hominids to other continents.

### Supplementary Information

Below is the link to the electronic supplementary material.

Supplementary file1 Supplementary Table S1 Prevalence of SNPs in the OAS locus in the Moroccan control cohort extracted from local WES database (XLSX 41 KB)

Supplementary file2 Supplementary Table S2 Demographic information, COVID-19 phenotype, genotype, and haplotype of three OAS1 SNPs of 146 individuals who were positive for SARS-CoV-2 (XLSX 19 KB)

Supplementary file3 Supplementary Table S3 Genotype of the Y-M81 marker in males who were positive for SARS-CoV-2 (XLSX 14 KB)

## Data Availability

All relevant data are within the manuscript and its Supporting Information files.
